# Multi-Locus Variable-Number Tandem Repeat Profiling of *Salmonella enterica* Serovar Typhi Isolates from Blood Cultures and Gallbladder Specimens from Makassar, South-Sulawesi, Indonesia

**DOI:** 10.1371/journal.pone.0024983

**Published:** 2011-09-15

**Authors:** Mochammad Hatta, Rob Pastoor, Pauline F. D. Scheelbeek, Andi R. Sultan, Ressy Dwiyanti, Ibrahim Labeda, Henk L. Smits

**Affiliations:** 1 Department of Medical Microbiology, Molecular Biology and Immunology Laboratory, Faculty of Medicine, Hasanuddin University, Makassar, South-Sulawesi, Indonesia; 2 Department of Digestive Surgery, Faculty of Medicine, Hasanuddin University, Makassar, South-Sulawesi, Indonesia; 3 KIT Biomedical Research, Royal Tropical Institute/Koninklijk Instituut voor de Tropen (KIT), Amsterdam, The Netherlands; Charité, Campus Benjamin Franklin, Germany

## Abstract

Multi-locus variable-number tandem repeat analysis differentiated 297 *Salmonella enterica* serovar Typhi blood culture isolates from Makassar in 76 genotypes and a single unique *S.* Typhi genotype was isolated from the cholecystectomy specimens of four patients with cholelithiasis. The high diversity in *S.* Typhi genotypes circulating in Makassar indicates that the number of carriers could be very large, which may complicate disease prevention and control.

## Introduction

Typhoid fever is a major disease problem in Indonesia and is endemic in most if not all major islands of the archipelago [1]. The causative agent of typhoid fever is *Salmonella enterica* serovar Typhi, a rod shaped, flagellated, aerobic, Gram-negative bacterium and exclusive human pathogen [2]. The genome of the pathogen which evolved only relatively recent in human history is highly conserved [3]–[5]. Different methods are available for the genotyping of *S.* Typhi of which single nucleotide polymorphism (SNP) [6], [7] and multi-locus variable-number tandem repeat analysis (MLVA) are most discriminatory [7]–[9]. Several studies have investigated the genetic diversity of *S.* Typhi at different locations in the Indonesian archipelago [6], [10], [11]. SNP was used to determine the phylogenetic relationship of *S.* Typhi isolates from a district of Jakarta on Java with isolates from other countries [6]. Using other typing methods supportive evidence was presented for the spread of *S.* Typhi genotypes throughout Indonesia [10] and for a rapid increase in the genetic diversity of *S.* Typhi in Papua New Guinea [11].

Typhoid fever is spread primarily through faecal contamination of food and drinking water by ex patients and carriers who secrete the pathogen with their faeces and urine [12]. The most common sites of infection are the ileum, liver, spleen, bone marrow and gallbladder. *S.* Typhi infection of the bile-rich gallbladder may result in a persistent local infection which then could lead to a chronic carrier state. With adequate treatment most typhoid patients clear the pathogen but nearly 3–5% is estimated to become carrier [13]. *S.* Typhi infection of the biliary tract is often accompanied with abnormalities such as inflammation and gallstones [14].

*S.* Typhi is one of the most important infectious diseases in South-Sulawesi and here were we report on the genetic diversity of the pathogen in Makassar, the capital city of Sulawesi. MLVA profiling of *S.* Typhi blood culture isolates revealed a large diversity of genotypes most of which were isolated once or only sporadically while others were found during several years. Four *S.* Typhi isolates obtained from gallbladder specimen of patients undergoing cholecystectomy had the same unique MLVA pattern distinct from that of all blood culture isolates indicating that specific *S.* Typhi genotypes may have a preference for infection of the biliary tree.

## Results

### 2.1. *S.* Typhi Genotypes of Blood culture Isolates of Typhoid Patients from Makassar

*S.* Typhi could be isolated from the blood of 57.1% of the typhoid patients. The male to female ratio of the *S.* Typhi blood culture positive patients was 0.92, the median age was 29 years (12–70 years), the mean duration of fever at the time of admission was 6 days (SD, 1.4 days) and the mean body temperature at admission was 38.5°C (SD, 0.5°C). The majority (94.0%) of the culture positive patient had a moderate disease condition with 6.0% suffering from severe disease.

**Figure 1:**
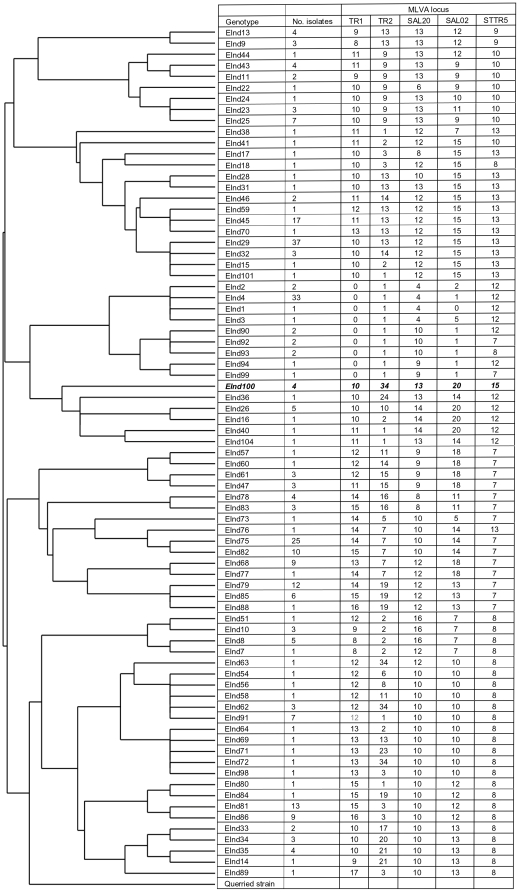
The EInd100 genotype and the dendrograph of *Salmonella enterica* serovar Typhi genotypes. Multiple-locus variable-number of tandem repeats analysis was performed for 297 *Salmonella enterica* serovar Typhi blood culture isolates from typhoid patients and four isolates from cholecystectomy specimens taken from cholelithiasis patients from Makassar, South-Sulawesi, Indonesia. Based on the variation observed at five highly variable loci a rooted tree was constructed by the unweighted-pair group method with an arithmetic mean algorithm using *S.* Typhi strain CT18 as reference (querried strain) and depicted together with the MLVA pattern and the number of isolates obtained from each genotype. Genotype EInd100 was isolated from four cholelithiasis patients; all other genotypes are from blood culture isolates from typhoid patients.

MLVA genotyping distinguished 76 *S.* Typhi genotypes among the 297 *S.* Typhi blood culture isolates obtained from typhoid patients from Makassar (Figure 1). The discriminatory power of the five tandem repeat loci used for genotyping ranged from *D* = 0.783 for STTR5 to *D* = 0.910 for TR2 (Table 1). The number of isolates of each genotype varied from one for 36 genotypes to 37 for genotype EInd29. With one exception (genotype EInd91), the fourteen genotypes that were isolated five times or more were all detected over a period of three to seven years (Table 2).

**Table 1:** Multi-tandem variable-number repeat genotyping of *Salmonella enterica* serovar Typhi isolates from Makassar.

Repeat^1^	No. of repeats	No. of alleles	*D* value (95% confidence interval)
STTR5	7, 8, 9, 10, 12, 13, 15	7	0.783 (0.743–0.824)
Sal20	4, 6, 8, 9, 10, 12, 13, 14, 16	9	0.784 (0.732–0.836)
TR1	0, 3, 8, 9, 10, 11, 12, 13, 14, 15, 16, 17	12	0.869 (0.838–0.901)
Sal02	0, 1, 2, 4, 5, 7, 9, 10, 11, 12, 13, 14, 15, 18, 20	15	0.899 (0.876–0.922)
TR2	0, 1, 2, 3, 5, 6, 7, 8, 9, 10, 11, 13, 14, 15, 16, 17, 19, 20, 21, 23, 24, 34	21	0.910 (0.884–0.935)

1 The nucleotide sequence of the repeats is GACCAT for STTR5, GCA for Sal20, GAAGAA for TR1, GTACCA for Sal02 and CCAGTTCC for TR2.

**Table 2:** Prevalence of *Salmonella enteric* serovar Typhi genotypes in blood cultures of typhoid patients in subsequent years in Makassar.

	Number of isolates with the specified *S.* Typhi genotype by year							
Genotype	2004	2005	2006	2007	2008	2009	2010	Total
EInd4				2	6	3	22	33
EInd8			1	2			2	5
EInd25				1	1		5	7
EInd26		1	1	1	2			5
EInd29	3	2	7	2	1		22	37
EInd45			1	1	6	5	4	17
EInd68		1	4	4				9
EInd75	3	2	7	4			9	25
EInd79	1		1				10	12
EInd81	1	3		1	4		5	14
EInd82		1		1			8	10
EInd85		1				3	2	6
EInd86	1	1			3		4	9
EInd91							7	7
Other	6	9	10	12	8	17	39	101
Total	15	21	32	31	31	28	139	297

### 2.2. *S.* Typhi Genotypes in Cholelithiasis Patients from Makassar

*S.* Typhi was isolates from gall tissue operation specimens of four (6.9%) cholelithiasis patients from Makassar (Table 3). Specimens from 49 (84.5%) patients were positive for other microorganisms and five (8.6%) cultures remained negative. The precise morphological location of the infections in the cholelithiasis patients could not be assessed as no attempt was made to seperate gallstones from tissue or gall fluid before starting the culture. As in most cholelithiasis patients the main complaints of the four patients with a *S.* Typhi infection included pain in the upper right quadrant of the abdomen with nausea and frequent vomiting; others presented with icterus of the conjunctiva or gastric pain. Two *S.* Typhi positive patients were females age 30 and 42 year and two were males age 24 and 42, respectively. All four patients were in the possession of an academic degree or had finished high school and belonged to the higher socio-economic class and denied ever been diagnosed with typhoid.

**Table 3:** Bacteria isolated from cholecystectomy specimens of patients from Makassar.

Organism	No. (%) of patients infected with each of the organisms
*Alkaligenes faecalis*	12 (20.7%)
*Staphylococcus epidermidis*	11 (19.0%)
*Staphylococcus aureus*	8 (13.8%)
*Candida sp.*	5 (8.6%)
*Salmonella* Typhi	4 (6.9%)
*Enterobacter agglomerans*	4 (6.9%)
*Bacillus subtilis*	4 (6.9%)
*Gram negative bacillococcus*	4 (6.9%)
*Acinetobacter calcoaceticus*	3 (5.1%)
*Klebsiella pneumoniae*	1 (1.7%)
None	5 (8.6%)

The four *S.* Typhi isolates obtained from the cholecystectomy specimens of the four cholelithiasis patients had the same MLVA pattern with 10 repeats for locus TR1, 34 repeats for TR2, 13 repeats for Sal20, 20 repeats for Sal02 and 15 repeats for STTR5 (Figure 1). This MLVA pattern had not been observed for any of the blood culture isolates and this genotype was designated *S.* Typhi genotype EInd100. The isolation from four cholelithiasis patient of a single *S.* Typhi genotype that is not present among the large diversity of genotypes cultured from the blood of typhoid patients is noteworthy. The EInd100 genotype was also not detected in blood culture isolates from typhoid patients from Papua and Kalimantan (unpublished observations). The EInd100 genotype showed a closest relationship with a group of five genotypes of which the MLVA pattern of genotype EInd36 was most similar to that of EInd100. The genetic distance between EInd100 and EInd36 was 19 compared with a distance of 57 calculated for the two least related genotypes EInd63 and EInd92 isolated from blood.

The four cholelithiasis isolates tested positive for the chromosomal *fliC* Hd flagellin gene and negative for the *fliC* Hj mutant flagellin gene, the *fljB^z66^* flagellin gene and the ind flagellin gene (not shown).

### 2.3. Geographic Location of the Homes of Cholelithiasis and Typhoid Patients

**Figure 2:**
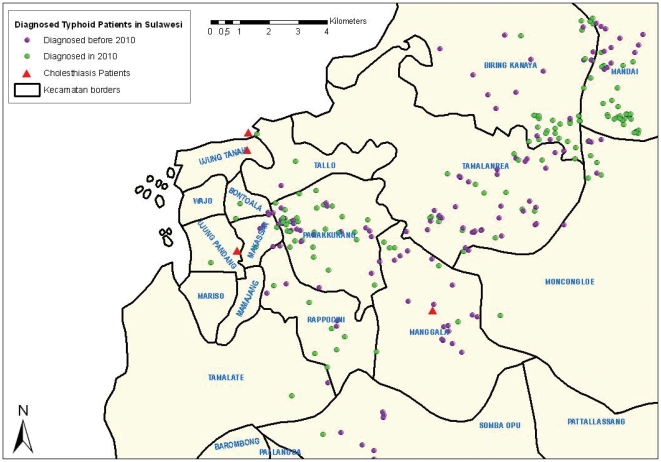
Geographic location of the homes of four cholelithiasis patients infected with *Salmonella enterica* serovar Typhi along with the location of all homes of culture positive typhoid patients. The location of the homes of the *S.* Typhi positive cholelithiasis patients are depicted on the map of Makassar along with the homes of all *S.* Typhi blood culture positive typhoid patients with patients diagnosed in 2010 and those diagnosed in the period 2004–2009 indicated with different colours.

The homes of three of the four *S.* Typhi positive cholelithiasis patients were located more closely to the city center of Makassar compared with the homes of the majority of the typhoid patients which were located mostly in more suburban and rural districts to the east of the city center (Figure 2). This seems to reflect the difference in socio-economic class between the cholelithiasis patients and the typhoid patients who predominantly belong to the middle and lower socio-economic groups. Surgery in Indonesia is in most cases restricted by the financial means of the patient.

## Discussion

MLVA profiling of *S.* Typhi blood culture isolates collected during a period of almost seven years from typhoid patients living in Makassar the capital city of Sulawesi in east Indonesia revealed a high diversity of genotypes with 76 genotypes detected in 297 patients. A comparison of the MLVA profiles with SNP based genotypes of a global population of *S.* Typhi isolates showed that by selecting highly polymorphic MLVA loci the former method has a much higher discriminatory power than SNP based genotyping [8] and is suitable for studying the diversity of strains circulating in a distinct geographic area. The five MLVA loci (TR1, TR2, Sal02, Sal20 and STTR5) used in the present study have high discriminatory values ranging from *D* = 0.783 for STTR5 to *D* = 0.910 for TR2. Expectedly, somewhat higher *D* values were reported for these loci in studies investigating *S.* Typhi in collections of isolates from different countries [8], [9].

While almost half (47.4%) of the genotypes isolated from typhoid patients from Makassar were isolated once or only sporadically other genotypes were detected during a period of at least several years. The large diversity in genotypes circulating in this city indicates that the number of carriers is likely to be very large but that transmission from most of these carriers is a rare event. Transmission may take place after fecal contamination of foodstuf and drinking water and may be limited by proper sanitation and by taking appropriate hygienic measures [12]. Consumption of food prepared by owners of open and mobile foodstalls practizing no or poor hygienic measures is very common in Makassar and food and drinks sold at such foodstall could function as major vehicles in the transmission of *S.* Typhi. Most inhabitants of Makassar take their drinking water from contaminated open water sources and the risk of contamination is increased by the use of open pit toilets. Contamination of a major food or water source by a carrier could promote the spread of a specific genotype.

In areas endemic for typhoid fever *S.* Typhi may be isolated from biliary tract specimens of patients with cholecystitis and cholangitis [15]–[17] and a persistent *S.* Typhi infection of the biliary tract may promote the formation of the carrier state. The chemical composition of the surface of the gallstones together with the specific milieu provide by the gall may promote the growth of *S.* Typhi on the surface of the gallstone [18]. The carrier state may then be induced by the further growth and persistence of the pathogen on the gallstone with the colonization of the gall and the formation of foci of infection in surrounding tissues including the gallbladder and duct, mesenteric lymph nodes and liver [19], [20]. To look for possible carriers we attempted isolation of *S.* Typhi from biliary tract specimens of patients undergoing cholecystectomy. *S.* Typhi was isolated from four cholelithiasis patients and remarkably the genotype deduced for all four isolates was identical (genotype EInd100) and differed from the genotypes of all blood culture isolates. Given the large diversity of *S.* Typhi genotypes detected in the blood of typhoid patients one might have expected to find different genotypes in the cholecystectomy specimens from different cholelithiasis patients. The four cholelithiasis patients belonged to the higher socio-economic class and might have ingested a particular food product or drink such as food or a drink served in a more exclusive restaurant contaminated from the same source. Also, the cholelithiasis patients who ranged in age from 24 to 42 year may have become infected at an early age when the EInd100 genotype was prevalent and a frequent cause of typhoid fever in the population of Makassar. Alternatively, but perhaps less likely, the four cholelithiasis patients may have been infected from a source outside Makassar or on another islands where the genotype is common. The more prevalent genotypes detected in the blood of typhoid patients were isolated over a period of at least several years but this does not exclude the possiblity that other genotypes may have prevailed in the past. The carrier stage may require months to develop but may persist for decades [21] and it is possible that the four cholelithiasis patients do not secrete the pathogen or that secretion by these patients does not lead to the contamination of a major food or water source. The population dynamics of *S.* Typhi has not been studied in detail and is likely to be influenced by a variety of factors including the presence and migration of carriers, the sanitation status and the risk for contamination of major water and food sources, and hygienic practices and the chance of infection. The fact that a *S.* Typhi strain that caused infection in several cholelithiasis patients is not detected in a series of typhoid patients enrolled during a period of several years indicates that changes in the dynamics of transmission can be considerably. The carrier of the *S.* Typhi EInd100 genotype who transmitted the pathogen to the four cholelitiasis patients may have ceased to secrete the pathogen or no longer contaminates a food or water source . Also, genetic drift may cause changes in the number of repeats but the timescale at which this takes place has not been investigated.

The finding of a single unique genotype in the cholecystectomy specimens may also imply that different genotypes through distinctive biological properties exhibit differences in tissue preference with the EInd100 genotype having an enhanced tropism for the biliary tract. MLVA loci are located in proximity of important genes such as the cell devision gene *ftsN* [7] and tandem repeat units may well be part of gene regulatory elements and differences in the repeat number could influence the functioning of such genes. Passage of *S.* Typhi through the human body and growth in the environment may require the rapid adaptation to harse adverse conditions and the colonization of the biliary tract may require adaptation to growth in a gall riche environment. Prolonged growth under such conditions could require adjustment of expression of a specific set of genes and could well favour selection of genetic variants with a specific MLVA pattern. Characterization of *S.* Typhi from biliary duct infections in patients from other socio-ecomic classes and from other locations may show whether indeed infection of gall, gall stones and surrounding tissue is confined to specific genotypes. Possibly, other genotypes infect the biliary tract but not in association with the presence of gallstones and the development of the specific symptoms characteristic for cholelithiasis. In Indonesia, surgery of the gallbladder and duct is offered by few hospitals including the hospital of the Hasanuddin University in Makassar and patients from other islands in the east Indonesian archipelago are refered to hospitals on Java.

A recent study by Crawford and coworkers [22] indicated that in *S.* Typhimurium the flagellum is required for attachment to and biofilm formation on cholesterol-coated surfaces. In *S.* Typhi the flagellum consists of the Hd flagellin antigen encoded by the *FliC* gene. Interestingly, certain *S.* Typhi strains circulating in Indonesia are unique by harboring either a mutant *Fli*C gene causing a deletion in the central core of the protein (the Hj antigen), and or the heterologous *fljB^z66^* or *ind* flagellin genes as a second flagellin gene [23]–[25]. Since carriers are important vehicles in the spread of *S.* Typhi and flagella could play a role in the establishment of the carrier state it was of interest to study the presence of these flagellin genes in the *S.* Typhi isolates obtained from the gallbladder specimens. All four cholelithiasis isolates tested positive for the *fliC* Hd flagellin gene and negative for the *fliC* Hj mutant flagellin gene, the *fljB^z66^* flagellin gene and the *ind* flagellin gene. We previously reported the presence of the *fliC* Hd flagellin gene in all and the *fljB^z66^* and *ind* flagellin genes in 14.5% of the blood culture isolates from South-Sulawesi [23]. The *fliC* Hj gene was not detected.

Gram-negative bacteria including *Escherichia coli* and *Klebsiella* spp. are among the most commonly isolated organisms from the bile and blood of patients with acute cholecystitis or cholangitis [15]–[17]. In our study the spectrum of organisms with as the more frequently isolated organisms being *Alkaligenes feacalis* and *Staphylococcus epidermidis* and *Staphylococcus aureus* was somewhat different from reports in the literature. Consistent with reports from typhoid fever endemic countries, *S.* Typhi was isolated from a minority (6.9%) of the cholecystectomy specimens.

In conclusion, we have detected a large diversity of *S.* Typhi genotypes in blood cultures of typhoid patients in a major city of east Indonesia and a novel *S.* Typhi genotype in gallbladder material of four cholelithiasis patients from the same city. The detection of a single unique genotype distinct from all *S.* Typhi blood culture isolates from typhoid patients in all four cholelithiasis patients is surprising and suggests that some *S.* Typhi genotypes may have a prefered tissue tropism. As the *S.* Typhi genotype isolated from the cholelithiasis patients was not isolated from any of the typhoid patients one may question the role of biliary tract infections in the transmission of all different *S.* Typhi genotypes. However, carrier state may take a long time to develop and the number of carriers could be very large and depending on the hygienic and sanitary conditions and the contamination of major food and drinking water sources few carriers may spread the pathogen to other individuals. Therefore, further studies are needed to confirm that specific *S.* Typhi genotypes are associated with gallbladder infections.

## Methods

### 4.1. Ethical Considerations

The project received ethical approval from the review board of the Department of National Education of the Hasanuddin University. Oral informed consent was obtained from the study participants after explanation of the procedure and the purpose of the study. Oral informed consent was applied as the collection of the specimens did not affect the surgical procedure to any extend and all clinical data was made anonymous before analysis. The collection of informed consent was witnessed by a nurse and or the medical officer in charge and was recorded on the medical file of the patient. The verbal consent procedure was approved by the ethical committee.

### 4.2. Patient Characteristics and Clinical Specimens

Gall tissue operation specimens were obtained from 58 cholecystectomy patients operated between March 2010 and March 2011 at the Digestive Surgery Department of the Dr. Wahidin Sudirohusodo Hospital. All patients were residents of Makassar, the capital of South-Sulawesi, Indonesia. The average age of the patients was 42.5 years (range, 24–72 years) and 22 (37.3%) were male and 36 (62.7%) female. The diagnosis was cholelithiasis in 42 patients, cholecystolithiasis and cholecystitis choledocholithiasis in six patients each, and cholecystitis and choledocholithiasis with obstruction of the icterus in two patients each. The main signs included gastric pain, abdominal pain in the upper right quadrant and icterus conjunctiva. Most of the patients suffered from nausea and frequent vomiting. None of the patients had signs and symptoms consistent with typhoid fever.

In 2010, *S.* Typhi blood culture isolates were obtained from 160 typhoid patients from Makassar and for the period 2004–2009 a further 149 archived *S.* Typhi blood culture isolates from typhoid patients from Makassar were available. Patients presented at different primary health care facilities (puskesmas) located in several districts of Makassar were included in the study.

A standard questionnaire was used to obtain socio-economic information and to appraise the sanitary situation at the household and hygienic practices of the patient.

### 4.3. Culture

Bacterial cultures of cholecystectomy specimens were performed by inoculating freshly collected operation material composed of gallstones, gall fluid and or gallbladder tissue into a Bactec plus aerobic/F bottle. Blood cultures were performed by the inoculation of 8 ml freshly collected venopuncture blood into the culture bottle. Culture bottles were transferred to the microbiology laboratory of the Hasanuddin University at the same day and placed in an incubator on arrival. Bacterial cultures were allowed to grow for 24 hours by incubation at 37°C after which 1 ml of broth was spread on a 9 cm diameter, 15 ml SS agar plate. After another incubation for 24 hours at 37°C plates were examined for colonies and individual colonies were picked and subject to biochemical identification.

### 4.4. Multiple-Locus Variable-Number Tandem Repeat Analysis

Five previously described MLVA loci designated TR1, TR2, Sal02, Sal20 and STTR5 were used for the genotyping of *S.* Typhi [8], [9]. Preliminary investigations showed that six other previously described loci exhibited no (TR3, TR4 and Sal10 with two repeats each) or a minimal (Sal15 with two alleles with one or two repeats, Sal06 with two alleles with four or five repeats and TR5 with three alleles with one, two or three repeats) variation only for isolates from east Indonesia and were not further used. All polymerase chain reactions (PCRs) were performed with the Platinium PCR Supermix kit (Invitrogen, Carlsbad, CA, USA). Each 25-µl reaction mixture contained 1 µl *S.* Typhi DNA, 5 pmol of each primer and 22.5 µl of the Supermix supplied with the kit. After an initial denaturation at 94°C for 2 min, the PCR reaction was performed for 35 cycles at 94°C for 30 s, 55°C for 30 s and 72°C for 1 min, followed by an extension step of 72°C for 7 min. Amplicons were sized on a 20 cm 2% agarose gel stained with ethidium bromide and viewed under UV illumination. To confirm the identity of the amplicons and to assess the copy number of the repeats the amplicons were purified using the QIAquick PCR purification kit and subjected to unidirectional sequence analysis. Genotyping was not successful for 12 blood culture isolates. The MLVA patterns obtained for the *S.* Typhi isolates were compared with the pattern of CT18, a serovar Typhi isolate isolated in 1993 in Vietnam. The cluster analysis was performed by the unweighted-pair group method with an arithmetic mean (UPGMA) algorithm, and a rooted tree was generated (http://minisatellites.u-psud.fr). In this study, we define a genotype as a strain with a distinct MLVA pattern. The distance between two genotypes is defined as the minimum number of changes in the number of repeats of any locus that converts one genotype to the other. MLVA analysis was not successful for two isolates from Kalimantan and three isolates from Sulawesi. The Simpson's index of diversity (*D* value) with 95% Confidence Intervals was calculated using an online tool (http://www.hpa-bioinformatics.org.uk/cgi-bin/DICI/DICI.pl).

### 4.5. PCR Detection of the Hd, Hj, z66 and Ind Flagellin Genes

The PCRs for the detection of the *fliC* Hd and Hj flagellin genes, the *fljB^z66^* flagellin gene and the *ind* flagellin gene were performed as described [23], [26].

### 4.6. Geographic Mapping

Spatial analysis was performed using the ArcGIS 9.2 program.
